# Combined Diagnostic Efficacy of Neutrophil-to-Lymphocyte Ratio (NLR), Platelet-to-Lymphocyte Ratio (PLR), and Mean Platelet Volume (MPV) as Biomarkers of Systemic Inflammation in the Diagnosis of Colorectal Cancer

**DOI:** 10.1155/2019/6036979

**Published:** 2019-01-17

**Authors:** Milica Stojkovic Lalosevic, Aleksandra Pavlovic Markovic, Sanja Stankovic, Mirjana Stojkovic, Ivan Dimitrijevic, Irena Radoman Vujacic, Daria Lalic, Tamara Milovanovic, Igor Dumic, Zoran Krivokapic

**Affiliations:** ^1^Clinic of Gastroenterology and Hepatology, Clinical Center of Serbia, Belgrade, Serbia; ^2^Faculty of Medicine, University of Belgrade, Belgrade, Serbia; ^3^Center for Medical Biochemistry, Clinical Center of Serbia, Belgrade, Serbia; ^4^Clinic for Digestive Surgery, Clinical Center of Serbia, Belgrade, Serbia; ^5^Clinic of Gastroenterology, Clinical Center of Montenegro, Podgorica, Montenegro; ^6^Mayo Clinic College of Medicine, Rochester, MN, USA; ^7^Mayo Clinic Health System, Eau Claire, WI, USA

## Abstract

**Background:**

Systemic inflammation in colorectal cancer (CRC) may be reflected by neutrophil-to-lymphocyte ratio (NLR), platelet-to-lymphocyte ratio (PLR), and mean platelet volume (MPV). This study was designed to investigate the efficiency of preoperative NLR, PLR, and MVP as a tool for the assessment of tumor characteristics in newly diagnosed patients with CRC.

**Patients and Methods:**

For 300 patients and 300 healthy volunteers, complete blood counts with automated differential counts were performed. The NLR was calculated by dividing the absolute neutrophil count by the absolute lymphocyte count; PLR was calculated by dividing the absolute platelet count by the absolute lymphocyte count. The diagnostic performance of NLR, PLR, and MVP was estimated by ROC curve.

**Results:**

ROC curve analysis showed high diagnostic efficacy of NLR and PLR in CRC patients with cut-off values of 2.15 (AUC = 0.790, 95% CI 0.736-0.884, Se = 74.1%, and Sp = 73%) and 123 (AUC = 0.846, 95% CI 0.801-0.891, Se = 73.5%, and Sp = 80%) compared to healthy controls, respectively. The diagnostic efficacy of three combined markers was superior compared with individual markers (AUC = 0.904, 95% CI 0.812-0.989, Se = 96%, and Sp = 70%).

**Conclusion:**

NRL, PLR, and MPV may be useful markers in diagnostic and early recognition of different stages of CRC; additionally combined all together have stronger diagnostic efficacy.

## 1. Introduction

Colorectal cancer (CRC) remains one of the most frequently diagnosed cancers in Western countries and one of the leading causes of cancer-related deaths, with an incidence of about 9%, estimated yearly [1]. According to the data of the Institute of Public Health of Serbia, CRC is the second leading malignancy after breast cancer in females and after lung cancer in males, in Serbian population, with about 3.400 newly diagnosed cases yearly [2]. Progress has been made in the setting of early diagnosing and treatment of patients with CRC; however, a large number of patients are still diagnosed in advanced disease stage. Colonoscopy remains the most efficient method for detecting CRC, yet its general application in the setting of screening is limited due to the uncomfortable experience and the high costs.

The most widely used screening biomarkers for CRC are fecal occult blood test (FOBT) and fecal immunochemical test (FIT); nevertheless, they can be affected by many dietary factors and have variable sensitivity due to its inability to distinguish upper and lower gastrointestinal bleeding [3, 4]. Carcinoembryonic antigen (CEA) and carbohydrate antigen (CA19-9) are commonly used biomarkers in everyday clinical practice for the detection and monitoring of CRC, but due to insufficient sensitivity and low organ specificity there is a need for more reliable biomarkers [5, 6]. Therefore, it is of a great importance to develop noninvasive, diagnostic, and treatment-predicting biomarkers. Present biomarkers are unable to predict biological behavior considering the fact that same-stage patients can have different clinical outcomes; thus, introducing novel markers could potentially be beneficial for personalized treatment of these patients [7]. Novel molecular diagnostics in the setting of CRC screening requests specialized equipment and trained personnel which are not widely available and it carries high economic burden [8]. Recent studies suggest that inflammation has a very important role in the process of carcinogenesis [9, 10]. Moreover, chronic inflammation affects all stages of tumor development. Several biomarkers are currently used to measure systemic inflammation such as C-reactive protein, neutrophil-to-lymphocyte ratio (NLR), and platelet-to-lymphocyteratio (PLR). Previous studies had shown that elevated NLR or PLR implies poor prognosis and/or survival for different cancer types, including CRC [11, 12]. Also, there are studies suggesting that systemic inflammation demonstrates unique behavior concerning CRC tumor stage development, which can be reflected by the levels of NLR and PLR [13]. Platelets are metabolically and enzymatically more active in systemic inflammation and have a higher prothrombotic potential. Mean platelet volume (MPV) determines platelet production rate and stimulation. Increased MPV has been noted in hepatocellular carcinoma, pancreatic carcinoma, and CRC [14].

The aim of this study was to investigate the individual and combined diagnostic accuracy of preoperative NLR, PLR, and MPV in newly diagnosed patients with CRC compared to healthy controls and also to investigate whether inflammatory markers could provide additional information regarding CRC phenotype characteristic. To our knowledge, this is the first study that investigates the combination of these three inflammatory markers in the setting of CRC.

## 2. Patients and Methods

### 2.1. Participants

Medical records of 300 newly diagnosed CRC patients were evaluated. Histological diagnosis of CRC was obtained from colonoscopy histology reports and later confirmed after surgical treatment. All patients were hospitalized in the period between May 2014 and March 2015 in the Clinic of Digestive Surgery, Clinical Center of Serbia, for surgical treatment of colorectal adenocarcinoma. While hospitalized, enrolled patients underwent abdominal ultrasonography, abdominal and/or pelvic CT/MRI when necessary, and chest radiography. In addition, patient's demographics, surgical details, postoperative pathological details, and postoperative outcome were also obtained. Patients were later staged according to TNM and Dukes classification [15]. The established study exclusion criteria were (a) recurrent CRC or 5-year history of another malignancy; (b) previous treatment with chemotherapy and/or radiotherapy; (c) evidence of other gastrointestinal, inflammatory, hematologic, hepatobiliary, pulmonary, and cardiovascular disease; and (d) treatment with antiaggregation and/or anticoagulant therapy, antilipemic therapy, and nonsteroidal anti-inflammatory drugs as well as recent blood transfusions.

As a control group, we analyzed medical records of 300 age-matched healthy volunteers with no significant statistical difference regarding sex, previously screened negative for CRC, who had done their annual health check-up. An informed consent was acquired from each patient before enrolling in the study. The Ethics Committee of our institution approved the study.

### 2.2. Methods

For all patients and healthy volunteers, complete blood counts (CBC) with automated differential counts were performed. Morning prior surgery, after 8-10-hour fast, peripheral blood (2 mL) samples were collected from the cubital vein. CBC analysis was performed in samples anticoagulated with EDTA within 4 hours after collection, using Coulter® LH750 Hematology Analyzer (Beckman Coulter, USA). The NLR was calculated by dividing the absolute neutrophil count (ANC) by the absolute lymphocyte count (ALC); likewise, PLR was calculated by dividing the absolute platelet count by ALC. Three levels of commercial control material (Beckman Coulter, USA) were run twice daily. During this study laboratory was included in Beckman Coulter's Electronic Quality Assurance Program (Hematology Interlaboratory Quality Assurance Program).

### 2.3. Statistical Analysis

Statistical analysis was carried out using the SPSS ver. 20.0 (SPSS Inc., Chicago, IL, USA). Patient's demographics and clinical and pathological characteristics were summarized descriptively. Continuous variables were expressed as mean ± standard deviation (SD). Normality of distribution was investigated by Kolmogorov-Smirnov test. The laboratory values between groups were analyzed using Mann-Whitney test. The clinicopathological variables among the groups were compared using Kruskal-Wallis test. Linear regression was used to investigate the relation between inflammatory markers, and binary logistic regression was used to combine inflammatory markers. The optimal cut-off values as well as sensitivity and specificity were determined according to the receiver operator characteristic (ROC) analysis. The best cut-off values were expressed using the Youden index. The area under the ROC (AUROC) curve also was calculated. A value of *P* < 0.05 was considered statistically significant.

## 3. Results

### 3.1. Clinicopathological Characteristics of CRC Patients

Demographic, clinical, and pathological characteristics of patients are summarized in Table 1. Of the 300 CRC patients, 164 (54%) were rectal cancer patients and 136 (46%) colon cancer patients. Furthermore, in 17 patients (6%), tumor had been localized in the caecum, in 14 (5%) in the ascending colon, 17 (6%) transversal, 20 (7%) descending, and 68 (22%) in the sigmoid colon, respectively. The distribution of patients in TNM stages I, II, III, and IV was 82 (27%), 74 (25%), 92 (31%), and 52 (17%), respectively. The patients with well, moderate, and poor tumor cell differentiation were 227 (76%), 63 (21%), and 10 (3%), respectively (Table 1).

### 3.2. Inflammatory Markers and CRC

CRC patients had significantly higher ANC and platelets compared to healthy controls and significantly lower ALC, MPV, and RBC (*P* < 0.01) (Table 2).

CRC patients had highly statistically significant different values of NLR and PLR compared to healthy controls (*P* < 0.01). Namely, CRC patients had significantly higher values of NLR and PLR compared to healthy controls (Table 2).

There were no statistically significant difference in values of NLR, PLR, and MPV regarding sex (*P* > 0.05), nor we have found significant correlation concerning age (*P* > 0.05).

**Figure 1:**
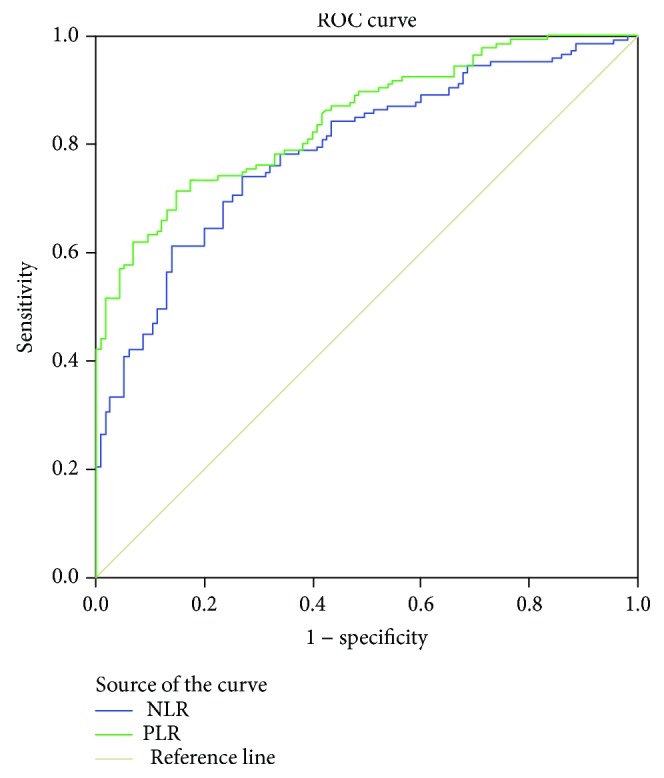
Diagnostic significance of NLR and PLR.

ROC curve analysis of NLR and PLR individually showed the best cut-off values of 2.15 (AUC = 0.790, 95% CI 0.736-0.884, Se = 74.1%, and Sp = 73%) and 123 (AUC = 0.846, 95% CI 0.801-0.891, Se = 74%, and Sp = 80%), respectively, for CRC detection (Figure 1).

ROC curve analysis for MPV had the best cut-off value of 8.5 (AUC = 0.816, 95% CI 0.764-0.869, Se = 74%, and Sp = 88%) (data not shown).

### 3.3. Combined Inflammatory Markers and CRC

**Figure 2:**
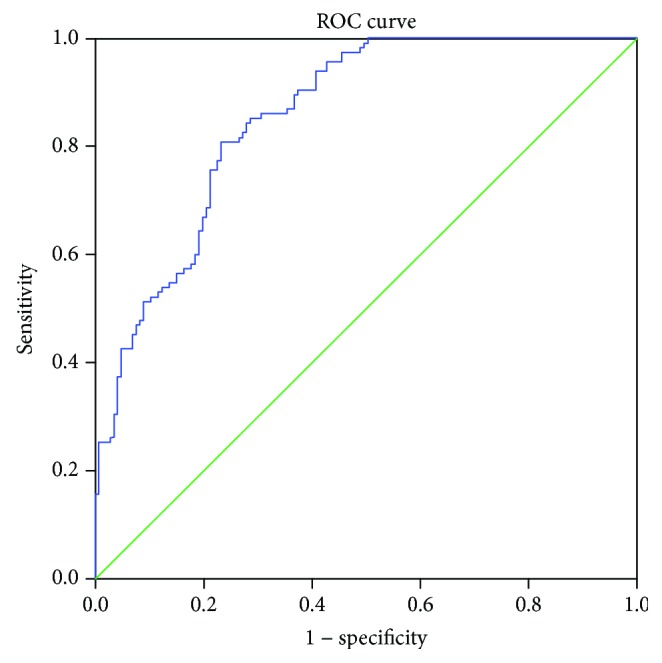
Combined diagnostic importance of NLR and PLR.

**Figure 3:**
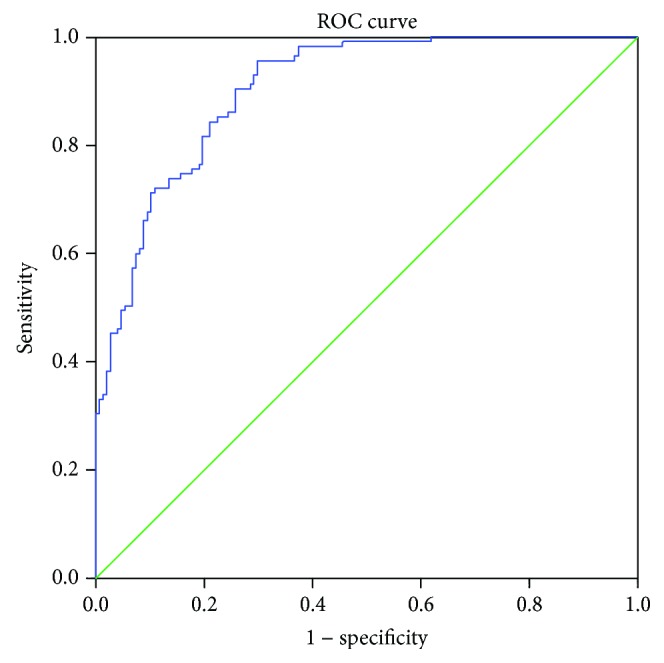
Combined diagnostic importance of NLR, PLR, and MVP.

We calculated AUC for combined determination of NLR and PLR, and it showed excellent diagnostic performance (AUC = 0.856, 95% CI 0.812-0.899, Se = 76%, and Sp = 80%) (Figure 2). Furthermore, when we combined MPV with NLR and PLR, diagnostic accuracy was even higher (AUC = 0.904, 95% CI 0.869-0.938, Se = 96%, and Sp = 70%) (Figure 3).

Also, we have found a positive association between NLR and PLR, which we demonstrated by regression equation *y* = 79.311 + 25.138*x* (*R*^2^ = 0.766; *P* < 0.01), and a significant negative association of MPV with both NLR and PLR (*r* = −0.281 and *r* = −0.488; *P* < 0.01), respectively (data not shown).

### 3.4. Inflammatory Markers and Tumor Phenotype Characteristics

NLR and PLR were significantly higher in all individual TNM stages (I, II, III, and IV) compared to controls (*P* < 0.01), and MPV values were significantly lower (*P* < 0.01) (Table 3). There was a significant difference between early stages (I, II) and more advanced disease stages (III and IV) in values of NLR and PLR (*P* = 0.023, *P* = 0.07, *P* < 0.05), while we found no significant difference in MPV values, although decreasing trend was observed through TNM stages I-IV (*P* = 0.662). Additionally, the combination of NLR, PLR, and MVP was significantly different in patients with advanced disease (IV) compared to other CRC patients (*P* = 0.04, *P* < 0.05).

Furthermore, Kruskal-Wallis test showed significant association of tumor extension with NLR (*P* = 0.04), with the increasing values through stages I to IV. However, Kruskal-Wallis test did not show significant association between PLR and also MPV and tumor extension (*P* = 0.19, *P* = 0.62, *P* > 0.05). There was no difference in NLR, PLR, or MPV values regarding tumor differentiation (*P* = 0.421, *P* = 0.383, *P* > 0.05).

## 4. Discussion

The role of systemic inflammation in the process of carcinogenesis has been widely studied and described previously [13, 16]. Namely, several studies have implied inflammation's critical role in CRC development and dysplasia formation, due to the induction of DNA damage by leucocyte-derived reactive oxygen species [17–19]. Moreover, cancer cells produce numerous inflammatory cytokines, leading to leucocyte tumor infiltration. In addition, inflammatory cytokines had been a subject of investigations concerning colorectal cancer cell proliferation, invasion, and metastases [20]. Itzkowitz and Yio in their study emphasize that chronic inflammation in inflammatory bowel disease patients leads to CRC development, without classical adenoma-carcinoma sequence [10]. Furthermore, in support of that assumption, Burr et al. emphasize that nonsteroidal anti-inflammatory drugs reduce the systemic inflammation and the risk of CRC and have been investigated in various precancerous lesions [21]. This supports the theory of Mariani et al. [22] that systemic inflammation is extremely significant especially in early cancer development. Therefore, NLR, PLR, and MPV as the markers of systemic inflammation potentially could be useful in evaluation of CRC patients.

Neutrophils represent a major leucocyte subclass, which promote cancer cell proliferation, angiogenesis, and metastasis by the production of proangiogenic chemokines and growth factors such as vascular endothelial growth factor (VEGF) and PK2/Bv8 [23]. However, lymphocytes produce cytokines, which inhibit the proliferation and metastatic spread of cancer cells, and provoke cytotoxic cell death [24]. It has been reported that in the process of carcinogenesis there is an increase in circulating neutrophils and consequential decrease in circulating lymphocytes [25]. A higher neutrophil count upstreams chemokine production: interleukin-1, interleukin-6 (IL-6), and tumor necrosis factor, therefore enabling tumor progression. Signaling pathways activated by IL-6 have been widely investigated in terms of CRC carcinogenesis in patients with inflammatory bowel disease [26]. In the study of Tang et al., blood concentrations of IL-6 were also correlated with CRC disease progression [27]. Considering this, NLR can reflect a balance between protumor and antitumor inflammatory status in CRC patients; therefore, the misbalance of neutrophils and lymphocytes can be associated with cancer progression.

Results of our study are in concordance with previously reported. Moreover, our results implied that CRC patients had significantly higher values of NLR compared to healthy volunteers which is also in accordance with previously published data [28]. Furthermore, NLR, PLR, and MPV values in CRC patients significantly differed from healthy controls, suggesting their potential role as noninvasive diagnostic biomarkers, which is also in concordance with studies of Peng et al. [29].

In the recent meta-analysis, Li et al. emphasized that elevated NLR values are associated with more advanced TNM stage and with poorer differentiation [30]. These findings are similar to our results, where we have found that NLR values were increasing through stages I-IV and furthermore significant difference in values of NLR and three combined markers when stage IV was compared to all other stages. In his study, Zahorec [31] showed a correlation of NLR with severity of clinical course in cancer patients, which is also in accordance with our results, presuming patients with more advanced disease could potentially have worse long-term prognosis.

The exact mechanism of platelets' role in the carcinogenesis process is not clearly elucidated. Till now numerous authors have attempted to investigate platelets' role in malignancies. In the study of Nieswandt et al., platelets have been noted as promoters of metastases, due to their ability to coat tumor cells making them unrecognizable for the natural killer cells produced by the immune system [32]. Likewise, one of the possible mechanisms is emphasized in the research of Gourban et al. where high levels of VEGF, PDGF, and PF4 were marked as possible clarification of elevated platelet count in patients with CRC, assuming that platelets could provoke new vessel development, as well as prevent bleeding from new vessels, leading to tumor cell promotion [26]. Additionally, Tang et al. reported that platelet VEGF was notably higher in cancer patients in comparison to healthy controls [27] and that could be a possible explanation for the increased number of platelets. Furthermore, platelets affect signaling pathways of DNA repair by the activation of epidermal growth factor receptor (EGFR) and DNA-dependent protein kinase [33].

PLR has been a subject of interest in a large number of studies concerning CRC patients and their outcome. Kilincalp et al. identified both NLR and PLR as poor prognostic factors [28]. Peng et al. suggested that both NLR and PLR could be used as early diagnostic markers in patients with CRC, which is in accordance with our results [29].

MPV as a marker of platelet size and activity is one of the most widely used markers. Additionally, MPV has been recognized as an inflammatory marker in cardiovascular, cerebrovascular, rheumatologic, and gastroenterological diseases [29–32]. Furthermore, MPV has been identified as an early diagnostic marker in the detection of gastric, pancreatic, hepatocellular cancer, and CRC [21, 34]. Regrettably, only a few number of studies have investigated MPV in patients with CRC, and the available data are still controversial. Our results showed that MPV can also be used as a diagnostic biomarker in CRC patients which is in concordance with the results of Wiesner et al. [35] but opposite to the results of Kilincalp et al. [28]. We can explain the discrepancies by the number of rectal cancer patients included in our study and higher BMI in the control group, considering that obesity also can affect MPV [33].

Most of the previously published studies have investigated markers of systemic inflammation independently and only a few of them combined two different markers [29]. To our knowledge, this is the first study to combine NLR, PLR, and MPV and has exceptionally high diagnostic accuracy. Moreover, this is the first study among Serbian population, and to our knowledge, the first study in this region of Europe, which is significant considering that regional prevalence of CRC is among the highest in Europe [36].

We are aware of the limitations of our study. It was a single-center study with all Caucasian subjects, and all three analyzed markers were in newly diagnosed patients, who already had some of the symptoms of CRC; hence, the effects of nonspecific inflammation could also exist and could influence the results.

In conclusion, this study supports the premise that inflammation is extremely significant in the process of CRC carcinogenesis. Additionally, combined diagnostic efficacy of NLR, PLR, and MPV could be a potential combination added to everyday CRC screening markers, considering it is currently a part of routine blood work analysis. Independent NLR, PLR, and MPV could be used daily as screening biomarkers in the detection of CRC even in early stages; however, NLR, PLR, and MPV combined all together have superior diagnostic performance.

## Figures and Tables

**Table 1:** Characteristics of CRC patients and healthy controls.

Variable	CRC (*n* %)	Healthy controls (*n* %)	*P* value
Age (mean ± SD)	61.63 ± 10.94	60.32 ± 12.21	>0.05
Gender (male/female)	160/140 (53%/47%)	150/150 (50%/50%)	>0.05
BMI	25.58 ± 3.47	26.13 ± 3.72	<0.05
*Location*			
Rectum	164 (54%)		
Colon	136 (46%)		
*Pathological differentiation*			
Well	227 (76%)		
Moderate	63 (21%)		
Poor	10 (3%)		
*T stage*			
1	52 (17%)		
2	42 (14%)		
3	142 (48%)		
4	64 (21%)		
*N stage*			
0	156 (52%)		
1	78 (26%)		
2	66 (22%)		
*Metastases*			
0	248 (83%)		
1	52 (17%)		
*Stage*			
I	82 (27%)		
II	74 (25%)		
III	92 (31%)		
IV	52 (17%)		

**Table 2:** Laboratory values of CRC patients and healthy controls.

	CRC	Healthy controls	*P* value (Mann-Whitney test)
WBC (mean ± SD) (10^9^/L)	7.20 ± 2.63	7.02 ± 1.76	>0.05
ANC (mean ± SD) (%)	4.89 ± 2.43	4.02 ± 1.55	<0.01
ALC (mean ± SD) (%)	1.52 ± 0.64	2.39 ± 0.76	<0.01
PLT (mean ± SD) (10^9^/L)	255.31 ± 77.97	218.48 ± 56.84	<0.01
NLR (mean ± SD)	4.80 ± 7.61	1.82 ± 0.83	<0.01
PLR (mean ± SD)	221.63 ± 209.47	97.51 ± 31.67	<0.01
MPV (mean ± SD) (fL)	7.61 ± 1.21	9.06 ± 1.41	<0.01

**Table 3:** Parameters of systemic inflammation in different TNM stages.

	Healthy controls	Stage I	Stage II	Stage III	Stage IV
NLR (mean ± SD)	1.82 ± 0.77	3.50 ± 0.46	3.81 ± 0.43	6.19 ± 1.3	6.47 ± 2.19
PLR (mean ± SD)	97.51 ± 2.95	175.72 ± 11.54	201.83 ± 21.55	254.30 ± 31.67	276.80 ± 58.54
MPV (mean ± SD)	9.10 ± 0.13	7.71 ± 0.18	7.68 ± 0.15	7.68 ± 0.17	7.20 ± 0.32

## Data Availability

The data used to support the findings of this study are available from the corresponding author upon request.
